# One-Dimensional Electron Transport Layers for Perovskite Solar Cells

**DOI:** 10.3390/nano7050095

**Published:** 2017-04-29

**Authors:** Ujwal K. Thakur, Ryan Kisslinger, Karthik Shankar

**Affiliations:** 1 Department of Electrical and Computer Engineering, University of Alberta, Edmonton, AB T6G 1H9, Canada; kissling@ualberta.ca (R.K.); Karthik.Shankar@nrc-cnrc.gc.ca (K.S.); 2 National Research Council, National Institute for Nanotechnology, 11421 Saskatchewan Drive NW, Edmonton, AB T6G 2M9, Canada

**Keywords:** photovoltaics, ordered bulk heterojunctions, solution processing, light scattering, surface traps, electrochemical anodization, solvothermal synthesis, metal oxide, TiO_2_, ZnO

## Abstract

The electron diffusion length (*L_n_*) is smaller than the hole diffusion length (*L_p_*) in many halide perovskite semiconductors meaning that the use of ordered one-dimensional (1D) structures such as nanowires (NWs) and nanotubes (NTs) as electron transport layers (ETLs) is a promising method of achieving high performance halide perovskite solar cells (HPSCs). ETLs consisting of oriented and aligned NWs and NTs offer the potential not merely for improved directional charge transport but also for the enhanced absorption of incoming light and thermodynamically efficient management of photogenerated carrier populations. The ordered architecture of NW/NT arrays affords superior infiltration of a deposited material making them ideal for use in HPSCs. Photoconversion efficiencies (PCEs) as high as 18% have been demonstrated for HPSCs using 1D ETLs. Despite the advantages of 1D ETLs, there are still challenges that need to be overcome to achieve even higher PCEs, such as better methods to eliminate or passivate surface traps, improved understanding of the hetero-interface and optimization of the morphology (i.e., length, diameter, and spacing of NWs/NTs). This review introduces the general considerations of ETLs for HPSCs, deposition techniques used, and the current research and challenges in the field of 1D ETLs for perovskite solar cells.

## 1. Introduction

The increasing global demand for energy has spurred research efforts to find new and improved sources of cheap, environmentally neutral, renewable energy. Inorganic solar cells based on materials such as crystalline silicon, cadmium telluride, or copper indium germanium selenide (CIGS) constitute mature technologies that exhibit a relatively high power conversion efficiency (PCE) of around 12%–20% in deployed modules [1] and thus dominate commercially available photovoltaic technologies. However, the relatively long energy payback times of inorganic solar cells [2,3] have partially impeded their pace to widespread deployment, and thus alternative approaches are being explored. Organic photovoltaics [4], dye-sensitized solar cells [5], halide perovskite solar cells [6], and quantum-dot solar cells [7] are examples of next generation solution-processable solar cell technologies that have emerged as lower cost, lower energy payback time alternatives to replace conventional solar cells [8,9]. Among these technologies, halide perovskite solar cells (HPSCs) are currently the topic of intense scientific and engineering interest due to their facile synthesis, use of earth-abundant constituent elements and high device performance [10]. A major breakthrough occurred in 2012 when Snaith et al. [11] reported an HPSC device with a PCE of 11% using a mixed halide perovskite layer (CH_3_NH_3_PbI_3−*x*_Cl*_x_*) as the active layer on nanoporous aluminum oxide. The first report of a HPSC was by Miyasaka and co-workers, who obtained a PCE of 3.8% in 2009 [12] using an active layer consisting of CH_3_NH_3_PbI_3_ (henceforth referred to as MAPbI_3_). 

**Figure 1:**
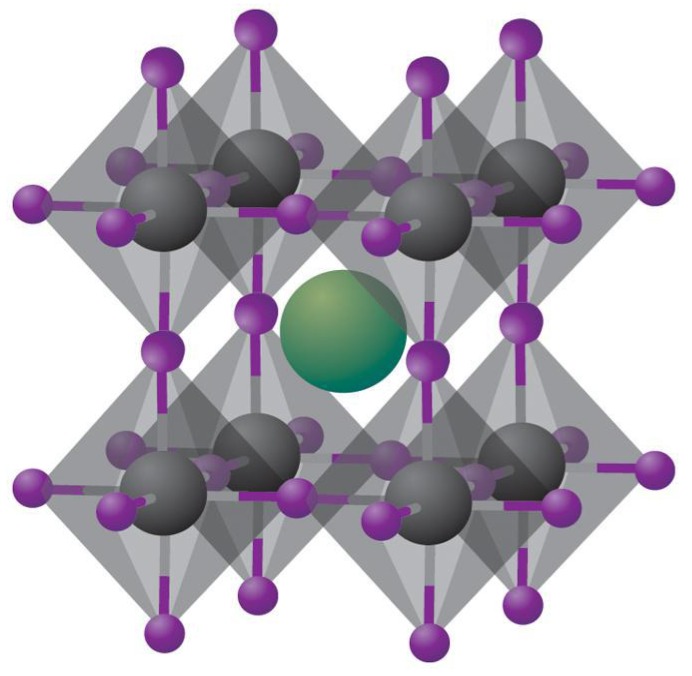
Schematic of cubic metal halide perovskites with the composition ABX_3_, with **A** = Univalent alkali metal cation (shown in green), **B** = Group IV metal cation (shown in grey) and **X** = halide ion (shown in purple). Reprinted with permission from Macmillan Publishers Ltd.: Nature Materials Ref. [14], Copyright 2014.

Perovskites, named after the Russian mineralogist Lev A. Perovski, are compounds having the molecular formula ABX_3_ and a specific crystal structure consisting of A and B cations together with X anions arranged in a cubic array of BX6 octahedra sharing corners, with the A cations placed in the cuboctahedral interstices which belong to the cubic Pm3m crystal structure as shown in Figure 1 [13]. In halide perovskites, X is a halide ion while A is a monovalent ion such as Cs^+^, CH_3_NH_3_^+^ (MA), or NH=CHNH_3_^+^, and B is a divalent ion such as Pb^2+^ or Sn^2+^. Unlike oxide perovskites, halide perovskites are not strictly ionic since the bond between the Group IV metal atom and the halide atom has some covalent character, which is maximum for iodide perovskites. 

Halide perovskites have unique properties [15] such as a direct optical bandgap, broadband light absorption, low carrier effective masses, dominant shallow point defects, benign grain boundaries, ambipolar transport, and long carrier diffusion lengths, due to which they have been investigated as light-absorbing and charge transporting materials in photovoltaic devices. In an astonishingly short period, this has led to an as-of-yet highest power conversion efficiency of 21.1% [16]. The highest efficiencies reported thus far, have been obtained using iodide perovskites, and mixed iodide-bromide perovskites. One challenge associated with perovskite solar cells is the choice of electron transport layer (ETL) in the solar cell architecture. It is important for the ETL to possess certain properties including an appropriate work function, high conductivity, fast charge transport, and a low recombination rate at the interface. Both inorganic and organic semiconductors have been used, with TiO_2_ being the most commonly used and having seen the most success. The morphology of the ETL layer is also of importance with planar film layers, mesoscopic particulate layers, and nanostructured layers being used. Planar film layers, while being the easiest to fabricate, are required to have a sufficient thickness in order to absorb all of the incident solar light. This thickness, however, is usually required to be longer than the diffusion length of electrons, which has been measured to be 100 nm or higher in iodide perovskites [17]. Mesoscopic electron transport layers have the advantage of allowing for infiltration of the perovskite, meaning that any dimension of the perovskite is kept to a minimum while still being able to absorb all the sun’s incident light [18]. However, it can be very difficult to completely fill the pore network of a mesoscopic structure with perovskite, and any unfilled areas inevitably lead to recombination at the exposed surfaces. In addition, a mesoscopic structure consisting of an interconnected network of nanoparticles, results in non-directional electron transport involving a random walk [19]. One dimensional nanostructures (1D-NS), however, are able to lever the same advantages of mesoscopic structures, while being able to allow for a more complete infiltration of perovskite into the electron transporting layer. Furthermore, their directional charge transport properties enable increased solar cell efficiencies [20,21,22,23]. In addition, 1D-NS with optimized geometries enable the improved management of incident photons in the solar cell. This review aims to summarize the fundamentals of preparing perovskite solar cells and how they relate to using one-dimensional electron transport layers (1D-ETLs), with emphasis on TiO_2_ and ZnO nanostructures as the leading candidates. The fundamentals of selecting and fabricating ETLs will be discussed, as well as the special considerations that one has to take into account when dealing with one-dimensional nanostructures. Finally, issues that still need to be solved in order for 1D-NS to achieve commercial viability are addressed.

## 2. Architecture and Working Mechanism of Devices

**Figure 2:**
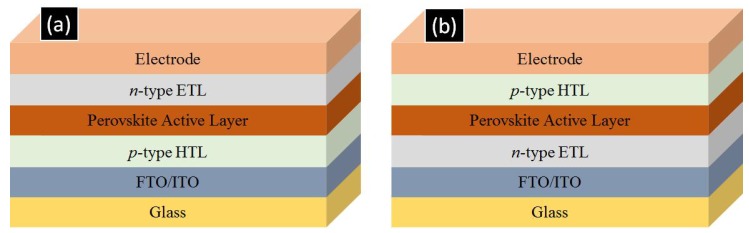
Layer configuration for (**a**) *p-i-n* type and (**b**) *n-i-p* type perovskite cell architectures. ETL and HTL refer to electron transporting layer and hole transporting layer respectively. FTO and ITO refer to fluorine tin oxide coated glass and indium tin oxide coated glass respectively

As shown in Figure 2, perovskite solar cells are fabricated in two major architectures, *p-i-n* and *n-i-p* type. In the *p-i-n* type architecture, a *p*-type hole transporting layer (HTL) such as CuO, NiO or PEDOT: PSS is deposited on a transparent conductive oxide (TCO) coated glass substrate namely fluorine doped tin oxide (FTO) or indium tin oxide (ITO) coated glasses. This is followed by the deposition of the perovskite active layer which is then coated over by a *n*-type film of [6,6]-phenyl-C_61_-butyric acid methyl ester (PCBM), ZnO, or C60 which acts as an ETL and subsequently a low work-function metal such as aluminum is evaporated to complete the device [24,25,26,27,28,29]. In the *n-i-p* type architecture, an *n*-type ETL such as TiO_2_, ZnO, SnO_2_ or WO_3_ is deposited on a TCO-coated glass substrate which is then followed by perovskite deposition. Over the perovskite, a *p*-type hole transporting layer (typically spiro-MeOTAD) is deposited and finally a high work function metal such as gold is deposited to complete the device [30,31,32,33,34,35,36,37,38]. This *n-i-p* configuration is necessarily used for the formation of solar cells involving 1D-ETLs to allow for proper infiltration of the perovskite into the ETL. Also of note is that ETL-free and HTL-free perovskite solar cells do exist but their photovoltaic performance is low [33,39].

## 3. Halide Perovskite Deposition Techniques

**Figure 3:**
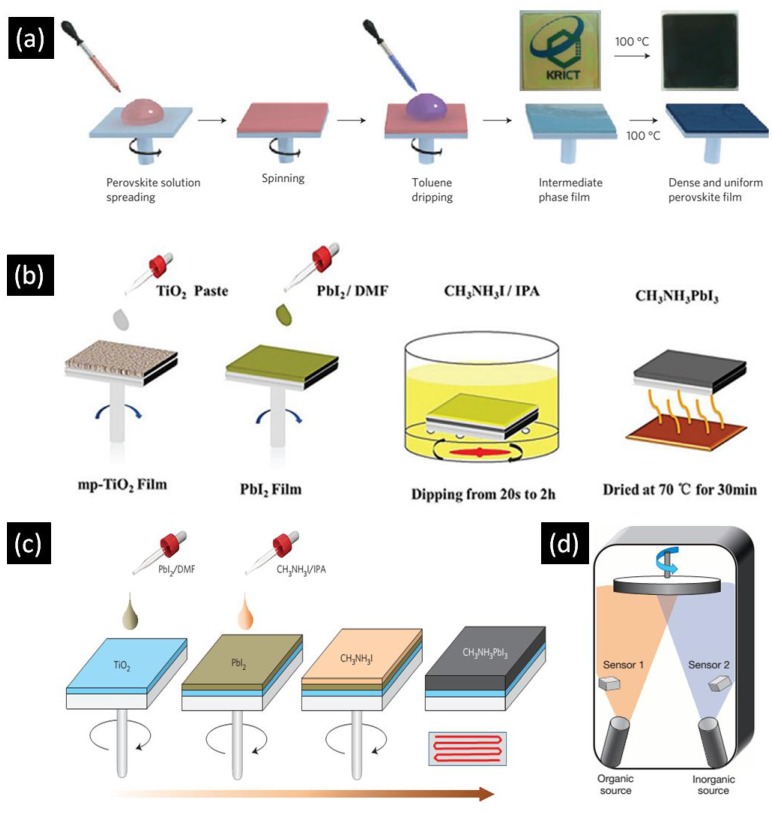
Schematic illustration of (**a**) one step spin casting (**b**) sequential deposition (**c**) two step spin casting and (**d**) dual source vapor deposition techniques for perovskite deposition. Adapted from Refs. [40,41,42,43] with permission from Macmillan Publishers Ltd. and The Royal Society of Chemistry.

The performance of perovskite solar cells is highly dependent on the crystal structure and morphology of the perovskite absorber, as well as the degree of contact the perovskite makes with the charge transport layers. These factors vary significantly in accordance with the deposition procedure, which should ensure good infiltration and contact when dealing with 1D-ETLs. Furthermore, to prevent direct contact between the electron transporting layer and hole transporting layer, an optimum thickness of perovskite overlayer is needed. This overlayer helps to ensure a sufficiently high value for the shunt resistance. Typically, single step spin coating, two step spin coating, and sequential deposition techniques are used to deposit the active layer in perovskite solar cells. Figure 3a is a block diagram of the one step spin casting method which involves the mixing of AX and BX_2_ in polar solvents such as γ-butyrolactone (GBL), dimethylformamide (DMF) and dimethyl sulfoxide (DMSO) to make the perovskite precursor solution. Spin casting of the precursor solution at sufficient revolutions per minute (RPM) is used to achieve the desired film thickness. This technique generally involves two spinning steps—one at low RPM and another at high RPM. In a typical synthesis, toluene or chlorobenzene is added to the spinning substrate prior to the completion of the second spinning step. After the spin coating step, the substrates are annealed in order to force the crystallization of the deposited perovskite layer. This procedure for perovskite active layer deposition was initially introduced by Miyasaka and coworkers in 2009 and perovskite solar cells with the highest PCEs reported to date employ this technique [12]. 

Figure 3b illustrates the methodology of the sequential deposition technique in which BX_2_ is dissolved in polar solvents such as DMF and DMSO while another solution of AX is made in 2-propanol. First, BX_2_ is spin coated over the substrate followed by calcination. After cooling down the substrate to room temperature, it is dipped into AX solution followed by annealing to crystallize the perovskite. The sequential deposition procedure was initially developed by Mitzi and colleagues in 1998 [44] while Gratzel et al. re-introduced this technique to fabricate perovskite solar cells in 2013 [45]. A major problem with this methodology is the length of time needed to convert BX_2_ into ABX_3_, during which some of the formed ABX_3_ can be leached away from the substrate. Two-step spin coating, illustrated in Figure 3c, is a modified version of sequential deposition in which BX_2_ is first spin coated over the substrate followed by calcination. After cooling down the substrate to room temperature AX dissolved in 2-propanol is spin coated over the dry BX_2_ layer. Another important deposition technique that has the ability to produce high performance perovskite solar cells is the dual source vapor deposition technique which was reported in 2013 by Snaith et al. [40]. As shown in Figure 3d, AX and BX_2_ are evaporated simultaneously from two different sources in a particular evaporation ratio to form ABX_3_ film on substrate which is then annealed. Other deposition techniques include sequential vacuum deposition [46], chemical vapor deposition [47], inkjet printing [48,49], spray coating [50,51], and slot die coating [52], but these methods have not been successful thus far in producing high efficiency photovoltaic devices. 

## 4. One Dimensional Nanostructures

1D-NS used in photovoltaics, taking the form of familiar structures such as rods, tubes, and wires, possess two dimensions of a size between 1 and 100 nm while the third dimension is typically in the range 200 nm–1 μm. A wide variety of top-down and bottom-up fabrication approaches exist for their synthesis, with varying degrees of complexity that allow for greater or lesser control over the final structure. Chemical synthesis strategies for 1D-NS are often the cheapest and least demanding in terms of deposition equipment, and include electrodeposition, sol–gel synthesis, solvothermal methods, and electrochemical anodization. While ease of fabrication and relatively high-throughput make these methods attractive options, they often suffer from the drawback of having a greater variability in final properties due to the indirect measures of control inherent to these methods. Strategies based on physical or physicochemical synthesis of 1D-NS such as vapor phase deposition, chemical vapor deposition, vapor-liquid-solid growth, and atomic layer deposition often result in superior electronic properties due to lower impurities and superior crystallinity while requiring dedicated deposition equipment and extreme conditions such as high vacuum and/or elevated temperatures. Even more precise control over the final structure can be obtained by techniques such as electron beam lithography or focused-ion beam writing or x-ray lithography, although these processes are much more expensive and of low-throughput.

**Figure 4:**
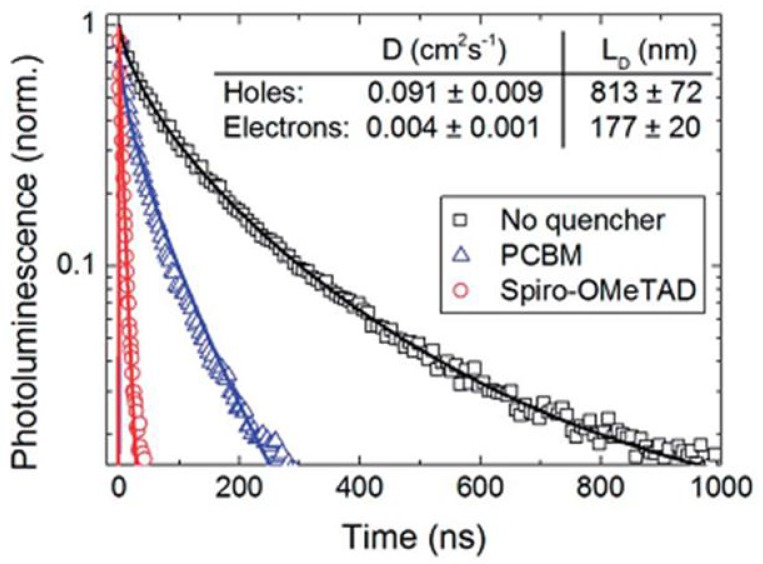
Time-resolved photoluminescence (along with stretched exponential fits) of MAPbI_3_ using the electron quencher [6,6]-phenyl-C_61_-butyric acid methyl ester (PCBM) shown as blue trianges or using the hole quencher layer Spiro-MeOTAD, shown as red circles. The data obtained without the use of a quencher, instead using insulating polymer poly(methylmethacrylate) (PMMA) is shown as black squares. Adapted from Ref. [53] with permission from The American Association for the Advancement in Science.

Several studies have found that the diffusion length of photogenerated electrons in halide perovskites is lower than that of photogenerated holes as shown in Figure 4, which calls for the application of nanostructured ETLs in perovskite solar cells [53,54]. 1D-NS offer a large surface area and the possibility of confinement of phonons and charge carriers, which leads to distinct electrical, optical and structural properties when contrasted with those of bulk materials. They may be used to limit the “random walk” of charges through a material; as there is only one direction in which charges may travel, the overall length of the path a charge takes on its way to being collected is reduced and thus charge recombination is limited. It is also important to use an optimized thickness of ETLs in perovskite solar cells (corresponding to the length of 1D ETL nanostructures). Because most perovskite semiconductors used in solar cells have a high extinction coefficient, an increase in the thickness of 1D ETLs does not generally improve the overall absorption of the device. However, increasing the thickness of 1D ETLs can reduce the photovoltaic performance of perovskite solar cells. The open circuit voltage decreases as the nanostructure length increases because of increased recombination at the ETL/perovskite interface [55,56,57]. The short circuit current too typically decreases because of ultraviolet photons absorbed by thicker ETLs which cannot be transmitted to the perovskite absorber layer [55,58]. The fill factor also reduces because of an increased series resistance of the solar cell. On the other hand, too thin an ETL does not provide a sufficient mesoscopic effect. If left unoptimized, forward- and back-scattering due to thick nanostructured ETLs can outcouple incident photons out of the device and reduce the amount of light harvested [59]. The nanorod packing density in 1D ETLs is another important parameter which plays a crucial role in the photovoltaic performance of solar cells. If the nanorods are densely packed, then there is less room between adjacent nanorods/nanotubes for the perovskite to be filled, resulting in poor filling and lower photovoltaic performance of the solar cells. Moderate and low density packing of the nanorods constituting the 1D ETL provide better filling and thus improved light harvesting efficiency [60,61,62]. Thus, it is important to optimize the thickness and morphology of 1D ETLs to take proper advantage of their directional charge transport and light management properties without harming other performance parameters of the solar cell. For high aspect ratio nanorods in the ETL, field emission of electrons into the perovskite is promoted, which in turn, may increase the dark current in the solar cells and thereby reduce the *V_oc_* value [59]. A superficial coating of perovskite is favored for high aspect ratio NRs with polar surfaces due to wetting considerations, as opposed to volumetric filling of the inter-rod spaces, which can exacerbate problems with extracting holes. 

## 5. Materials for One Dimensional Electron Transport Layers

**Figure 5:**
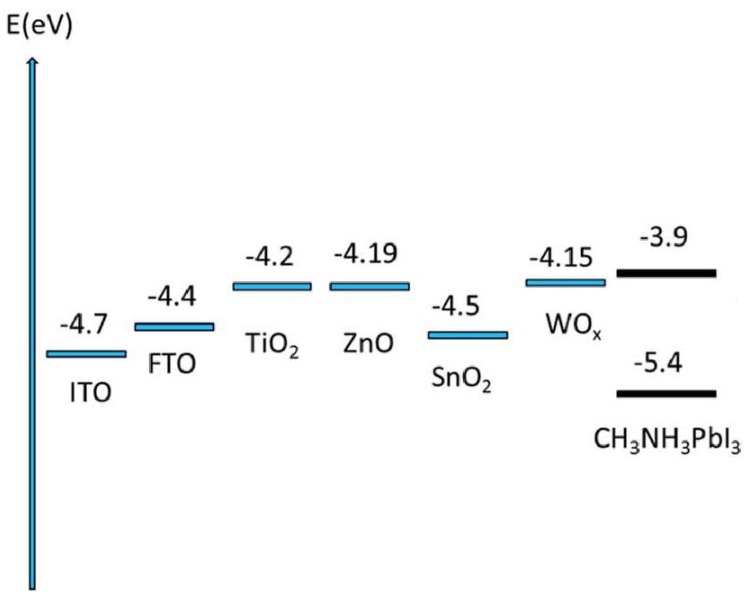
Energy level diagrams illustrating the position of the conduction band minimum vs. the vacuum level in various electron transport layer materials. Adapted from Ref. [77] with permission from The Royal Society of Chemistry.

Both inorganic and organic semiconductors have been used as electron transport materials, although it is inorganic materials that have received attention for their applicability as one-dimensional nanostructures since the morphological integrity of organic semiconductor nanostructures typically does not survive the subsequent solution deposition of the halide perovskite due to the partial or complete solubility of the organic semiconductors in solvents such as GBL, DMF, and DMSO [63,64]. It is worth noting, however, that research efforts towards the development of one-dimensional organic semiconductors are being made through techniques such as solution-phase synthesis, templating, electrospinning, and nanolithography [65]. TiO_2_, ZnO, SnO_2_, and WO*_x_* are the most commonly used materials (energy level diagram shown in Figure 5) for 1D-ETLs, although other inorganic semiconductors exist that could potentially be used in a one-dimensional nanostructure, such as Zn_2_SnO_4_ [66], BaSnO_3_ [67], and SrTiO_3_ [68]. While not a focus of this review, common organic electron transport materials include fullerenes, methanofullerenes, and perylene derivatives. There are several major considerations when selecting the material to use as ETL, including the energy level alignment with respect to the particular perovskite absorber used, the doping density, the density and energetic depth of trap states in the material, the electron mobility, and hole blocking action. The material most commonly used as the ETL is TiO_2_, which exhibits good electron transporting properties and is known to be non-toxic and chemically stable. TiO_2_ exists in three crystalline phases—anatase, brookite, and rutile [69], among which the anatase phase TiO_2_ has been used the most widely and achieved the highest performance in photovoltaic applications due to possessing a higher effective surface area, faster electron transport, and longer electron lifetime than the other two phases [70,71]. The anatase phase of TiO_2_ also exhibits exceptional hole blocking, which is highly desirable. However, a major issue with TiO_2_ and its potential to be used in commercial applications, is the high temperature processing typically required to anneal the TiO_2_ into these crystalline forms, since the as-fabricated TiO_2_, is often amorphous. In light of this problem, efforts have been made to develop crystalline TiO_2_ without the need for high-temperature processes [72]. Compared to other candidate ETL materials, TiO_2_ possesses a high density of shallow- and deep-trapping states [73] that in turn promote Shockley-Read-Hall type recombination and Fermi-level pinning at interfaces with other materials. The most widely investigated replacement for TiO_2_ is ZnO, which has a 5–10 fold higher electron mobility [74] and suitable energy levels. However, processing issues and the poorer chemical stability of ZnO have hindered the use and development of ZnO as an ETL. SnO_2_ has an electron mobility nearly two orders of magnitude higher than that of TiO_2_ [75], yet shows much lower PCEs due to non-optimal energy levels and high recombination rates. Similarly, WO*_x_* has exhibited poor performance when used by itself. However, both SnO_2_ and WO*_x_* have shown increased performance when used in combination with ZnO and TiO_2_, such as in a core shell structure [38]. Such a core-shell structure can effectively suppress recombination and improve the electron transfer at the ETL/perovskite interface, resulting in improved solar cell performance [61,76].

## 6. Classes of One Dimensional Nanostructures Used as ETLs in Perovskite Solar Cells

The vast majority of 1D-ETLs take the form of nanotubes or nanorods (also known as nanowires). However, other morphologies do exist, including nanofibers and core-shell structures utilizing two or more materials as the electron transporting layer. Herein, we focus on the structure and function of TiO_2_ nanotubes, TiO_2_ nanorods, and ZnO nanorods, with the reported solar cell performance of TiO_2_ 1D-ETLs and ZnO 1D-ETLs summarized in Table 1 and Table 2 respectively. Table 3 and Section 6.4 summarize unorthodox 1D-ETL morphologies and materials.

### 6.1. TiO_2_ Nanotube Arrays

Vertically oriented, self-organized, highly ordered TiO_2_ nanotubes (NTs) have attracted interest for use in dye-sensitized solar cells, quantum dot-sensitized solar cells, and bulk heterojunction photovoltaic devices since the mid-2000s [78,79,80,81,82]. While various methods to fabricate TiO_2_ nanotubes exist—including sol–gel [83], atomic layer deposition into nanoporous templates [84], and hydrothermal methods [85]—by far the simplest route to obtain highly uniform TiO_2_ nanotube arrays is the anodization method. The anodization method involves applying a sufficiently anodic voltage to Ti metal foils or Ti thin films vacuum deposited on to a TCO-coated glass or plastic substrates in an electrochemical cell with an appropriately selected electrolyte [86,87,88]. The key to the process is the simultaneous oxidation of Ti to form a TiO_2_ oxide layer along with the electrochemical/chemical dissolution of TiO_2_ in the form of pitting, a feat accomplished by the presence of anions such as F^−^, Cl^−^ or ClO_4_^−^ in the electrolyte [89,90,91,92]. While the exact formation process is not fully understood and remains contentious [93,94,95,96], the end result is that an entire TiO_2_ nanotube array is typically formed all across the Ti film over the course of minutes or hours. Depending on the conditions used and the thickness of the precursor Ti film, these nanotubes have lengths ranging from a few hundred nanometers to several hundred micrometers, with pore diameters ranging from tens to hundreds of nanometers [97,98,99]. For perovskite solar cells, a nanotube length (ETL film thickness) of <1 μm is preferred in order to match the penetration depth of radiation in the active layer. As-formed, the titania nanotubes are amorphous, and they are annealed at temperatures of 350–650 °C in air or flowing oxygen to produce *n*-type rutile or anatase phase TiO_2_ nanotubes [100]. There are also reports of using suitable electrolyte recipes during electrochemical anodization and/or post-anodization treatments to induce a strong preferential crystallographic texture in order to form single crystal-like TiO_2_ nanotube arrays [101,102]. Polycrystalline anatase-phase nanotubes have been predominantly used in HPSCs to date (see Table 1 for a concise summary of the performance obtained from the reported use of TiO_2_ nanotubes and nanowires in perovskite solar cells). 

**Figure 6:**
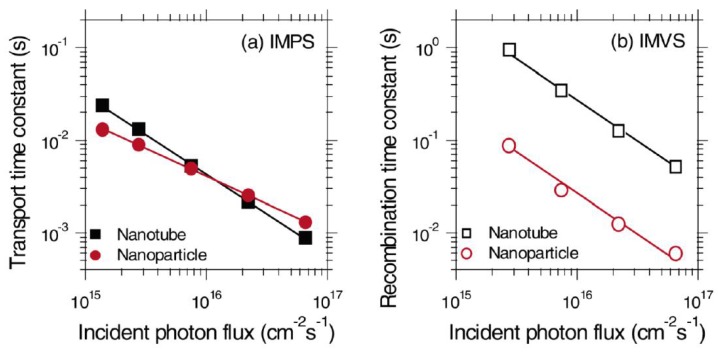
(**a**) IMPS and (**b**) IMVS plots of TiO_2_ nanoparticle and NT based DSSCs to calculate transport time and recombination time respectively. Reprinted with permission from [20]. Copyright 2007 American Chemical Society.

The photogenerated electron transferred to the electron transporting layer can itself recombine with the hole in the perovskite layer. Such a back-electron transfer process provides a second recombination pathway in addition to geminate recombination in the perovskite; thus in order to enhance the photovoltaic performance of a solar cell, the electron transport layer must offer faster charge transport rate compared to the recombination rate due to back-electron transfer and hence must achieve a high charge collection efficiency. Compared to the nanoparticle-based ETLs, one-dimensional nanostructured ETLs have the potential to facilitate a higher charge transport rate and lower the rate of recombination [103,104]. Zhu et al. used intensity modulated photocurrent spectroscopy (IMPS) and intensity modulated photovoltage spectroscopy (IMVS) to compare the transport and recombination time constants of TiO_2_ nanoparticle- and NT-based dye-sensitized solar cells (DSSCs) as shown in Figure 6 [20]. It was shown that recombination in NTs was almost 10 times slower compared to that in nanoparticle films, while the transport time in both structures was almost the same. Thus, the resulting charge collection efficiency of NTs was 25% better compared to nanoparticulate ETLs, allowing the use of thicker NT films to enhance absorption and improve the light harvesting efficiency. Similarly, in the case of perovskite solar cells, an optimized length of TiO_2_ NTs provides for an increased electron lifetime, low charge recombination rate, and higher charge collection efficiency which in turn offer the potential for improved photovoltaic performance of NT-based perovskite solar cells compared to nanoparticulate ETLs [105].

**Figure 7:**
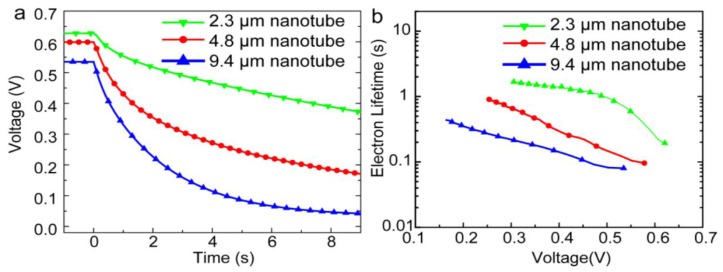
(**a**) Open circuit voltage decay plots and (**b**) electron recombination lifetime vs. voltage plots of MAPbI_3_ perovskite solar cells with different lengths of TiO_2_ NTs as the ETL. Reprinted with permission from Ref. [57], published by the Royal Society of Chemistry.

In 2014, Gao et al. [57] reported a perovskite-sensitized solar cell with a PCE of 5.52% using TiO_2_ NTs as the ETL for the first time. They used a two-step anodization process of Ti foil to form free standing TiO_2_ NT membranes. By keeping the voltage constant and increasing the anodization time they could form free standing TiO_2_ NTs of different lengths (2.3 µm, 4.8 µm, and 9.4 µm) which were transferred to a compact TiO_2_-coated FTO substrate. After casting MAPBI_3_ on the TiO_2_ NTs, they used an iodide-based liquid electrolyte to complete the solar cell. As shown in Figure 7, the photovoltage in shorter NTs was found to decay more slowly compared to longer NTs, which indicated that longer NTs have a shorter electron lifetime and a higher charge recombination rate, resulting in a lower conversion efficiency. In a recent report [106], they ground down TiO_2_ NTs to make discrete NTs and dissolved them in terpineol and ethanol. They subsequently spin coated this solution over a compact-TiO_2_ film on a FTO substrate to obtain a NT network as the electron transport layer. They were able to achieve a short circuit current density of 24.8 mA·cm^−2^ which is the highest recorded at present when using MAPbI_3_ as the active layer. The maximum PCE that they measured was 13.8% with a *V_oc_* and FF of 0.88 V and 0.63 respectively [106]. 

**Figure 8:**
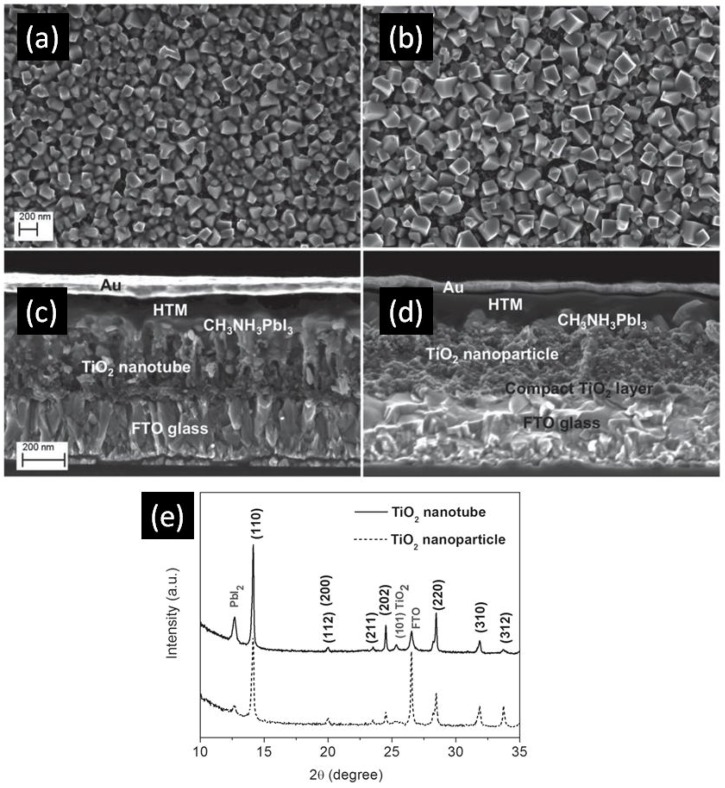
Scanning electron microscopic (SEM) images of MAPbI_3_ deposited on (**a**) TiO_2_ nanotubes and (**b**) TiO_2_ nanoparticles, and cross-sectional images of a complete photovoltaic device using (**c**) TiO_2_ nanotubes and (**d**) TiO_2_ nanoparticles; (**e**) X-ray diffraction (XRD) spectrum displaying the incomplete conversion of PbI_2_ using TiO_2_ nanotubes. Reused with permission from Ref. [105]. Copyright 2015 Wiley and Sons.

**Figure 9:**
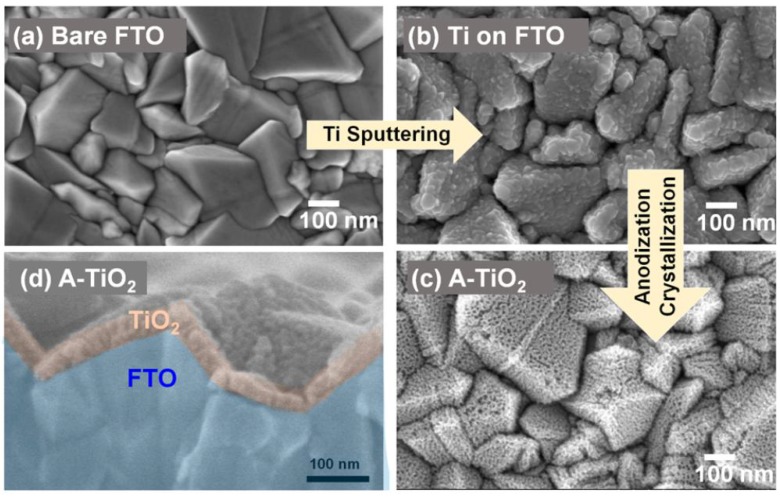
Top SEM image of (**a**) bare FTO (**b**) Ti sputtered on FTO (**c**) anodized TiO_2_ and (**d**) cross-sectional SEM image of anodized TiO_2_. Reprinted with permission from Ref. [107]. Copyright 2016 American Chemical Society.

In 2014, Qin et al. directly formed TiO_2_ NTs by anodizing a Ti-coated FTO substrate to obtain TiO_2_ NTs with lengths of 400–450 nm, pore diameters of 55–85 nm and wall thicknesses of 15–20 nm [105]. The perovskite used was MAPbI_3_ which was infiltrated into the titania nanotube ETL by the solution-based sequential deposition method. Next, the hole transporting layer and gold contacts were deposited on top of the perovskite layer as shown in Figure 8c,d. Using these NTs as the ETL, they were able to achieve a PCE of 14.8%. In this report [105], perovskite could be effectively infiltrated into both the inside of the nanotubes as well as the inter-tubular spaces, allowing for effective charge transfer between the two materials. Furthermore, the use of a single-end open nanotube array ETL meant that the perovskite remained relatively sealed from external moisture, a problem that often occurs in TiO_2,_ nanoparticle-based solar cells unless additional sealing is used. Nevertheless, issues with the use of perovskite were found. Incomplete conversion of PbI_2_ to CH_3_NH_3_PbI_3_ occurred, especially when compared to TiO_2_ nanoparticles, as shown in the XRD spectrum in Figure 8e. The perovskite crystal size was also found to be smaller when deposited on nanotubes compared to nanoparticles, as shown in Figure 8a,b, this being an issue because a smaller crystal size results in a larger number of grain boundaries with concomitant increase in carrier trapping and charge recombination rate. Improved perovskite deposition methods and optimized nanotube dimensions could ameliorate these problems and lead to considerably higher efficiencies. The highest PCE of perovskite solar cells with TiO_2_ NTs as the ETL at present is 15.2%, reported by Choi et al. [107]. They found that HPSCs using 40 nm-thick highly ordered single crystal-like TiO_2_ nanopores obtained by anodizing sputtered Ti gave a better photovoltaic performance compared to HPSCs using spin-coated compact TiO_2_ films because of the high contact area between anodic TiO_2_ and perovskite as well as the superior electron transport properties of anodic TiO_2_ NTs. Figure 9 shows the morphology of this one-dimensional TiO_2_ layer.

Wang et al. demonstrated backside illuminated flexible perovskite solar cells by using anodized TiO_2_ NTs on Ti foil as the ETL with a PCE of 8.31% which was the highest reported PCE of flexible perovskite solar cell at that time [108]. A major problem limiting PCEs with backside illuminated solar cells is the strong absorption of ultraviolet and violet photons by the HTL, thus leading to low external quantum efficiencies for wavelengths in the range 359–425 nm [108]. Similarly, Salazar et al. demonstrated HTL-free perovskite solar cells with anodized TiO_2_ NTs on FTO as the ETL with a PCE of 5% [109].

### 6.2. TiO_2_ Nanorod Arrays

**Figure 10:**
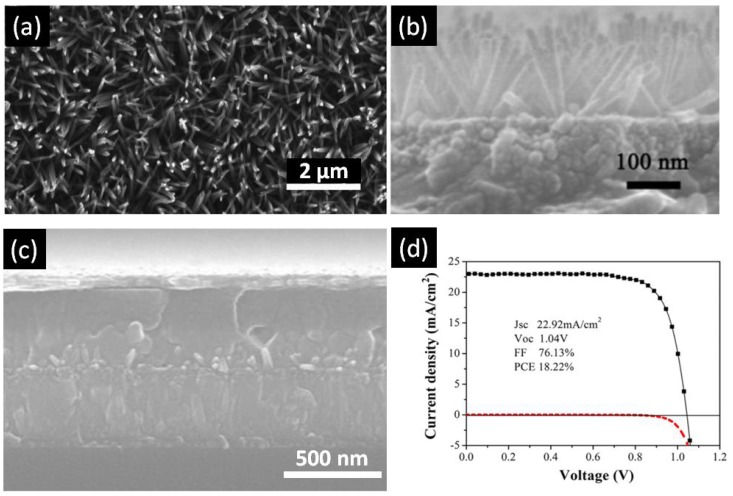
(**a**) Top-view SEM image of TiO_2_ nanowires; (**b**) Cross-sectional SEM image of TiO_2_ nanowires; (**c**) Cross-sectional SEM image of perovskite solar cell fabricated using a TiO_2_ nanowire array as the ETL; and (**d**) *J–V* curve of the best performing perovskite solar cell with TiO_2_ nanowire array ETL. Adapted with permission from Ref. [116]. Copyright 2016 American Chemical Society.

TiO_2_ nanorods (NRs) are another popular configuration for ETLs in perovskite solar cells (summarized in Table 1). TiO_2_ nanorods are another popular one-dimensional nanostructure that has attracted tremendous interest as ETLs in perovskite solar cells (summarized in Table 1). Among the various methods to grow TiO_2_ NRs—such as sol–gel processes [117], chemical vapor deposition [118,119,120], vapor-liquid-solid growth [121], and pulsed laser deposition [122]—hydro/solvothermal synthesis is the simplest method to obtain high quality TiO_2_ NRs [123]. Hydrothermal synthesis refers to a heterogeneous reaction occurring under high pressure and temperature in the presence of aqueous solvents to dissolve and subsequently recrystallize the material that under ordinary conditions would be insoluble [112,124]. Solvothermal methods are similar to hydro-thermal methods except the solvent used is non-aqueous [125]. Most highly efficient perovskite solar cells have the TiO_2_ nanowire ETLs grown by hydrothermal synthesis, which has the added advantage of being directly synthesizable on rutile-phase FTO-coated (amorphous) glass substrates [125]. Unlike the anodic TiO_2_ nanotubes, TiO_2_ nanorods fabricated by the hydrothermal process are composed of the rutile phase of TiO_2_ [123], which exhibits inferior electronic properties in comparison to the anatase phase of TiO_2_. Even though anatase nanorod arrays have been used in dye-sensitized solar cells, their synthesis is less facile compared to rutile nanorod arrays which may be why their use in HPSCs has not been prominent. The length and diameter of the solvothermally grown rutile nanorod arrays are controlled by synthesis conditions, such as temperature, pressure, precursor solution composition, pH, and reaction time. Hydro/solvothermal synthesis provides flexibility in being able to tune the dimension and aspect ratio of TiO_2_ NRs just by changing the growth time and concentration of precursor. Qui et al. was first to use TiO_2_ nanorods as an ETL with perovskite as the light absorber [110]. Using a thin layer of MAPbBr_3_ on 1.5 µm tall nanorods, they reported a PCE of 4.87% [110]. This report triggered new research on application of TiO_2_ nanorods as an electron transport layer for perovskite solar cells. Park et al. [111] compared performance of longer and shorter TiO_2_, nanorods. Though they could not find any considerable difference in recombination resistance between longer and shorter nanorods, they concluded that shorter nanorods could provide better infiltration of perovskite. By using MAPbI_3_ as a light absorber, they were able to achieve a PCE of about 9.4% [111]. In 2014, Jiang et al. [112] fabricated a perovskite solar cell using 900 nm-long TiO_2_ nanorods as the ETL and achieved an efficiency of 11.7%, a record for TiO_2_ nanorods at that time. The higher efficiency was attributed to wide-open voids between the nanorods into which perovskite was able to effectively infiltrate [112]. In 2012, the same group reported on the faster electron transport capabilities of TiO_2_ nanorods compared to mesoscopic TiO_2_ [21]. Subsequently, several devices using this structure were reported, having a PCE lower than 15% [56,126,127]. Subsequently, Hong et al. were able to demonstrate in 2015 a TiO_2_-nanorod based perovskite solar cell with an efficiency of 13.5%, achieved by passivating the surface of the nanorods with a thin layers of TiO_2_ in an attempt to limit and grain boundaries surface traps [115]. Most recently, in 2016, Li et al. were able to achieve record efficiencies of 18.22% [116], shown in Figure 10. In this work, the diameter and lengths of the nanorods used were around 2–40 nm and 180 nm respectively and were synthesized from a precursor containing 2-ethyl-butyric acid. They were able to achieve excellent perovskite deposition, and in so doing, ensured no contact between the TiO_2_ and the hole transporting layer. Furthermore, they utilized a UV-ozone cleaning process to eliminate organic residues on the nanorod surface, strengthening the contact between perovskite and TiO_2_ and thus decreasing surface traps and increasing the efficiency. 

### 6.3. ZnO Nanorod Arrays

ZnO is an *n*-type semiconductor with band gap of 3.37 eV and a higher electron mobility than TiO_2_ [128,129]. This results in less recombination loss in ZnO compared to TiO_2_, which makes ZnO superior in balancing the charge transport in perovskite. The reported photovoltaic performance of perovskite solar cells fabricated using ZnO NRs as the ETL is summarized in Table 2.

**Figure 11:**
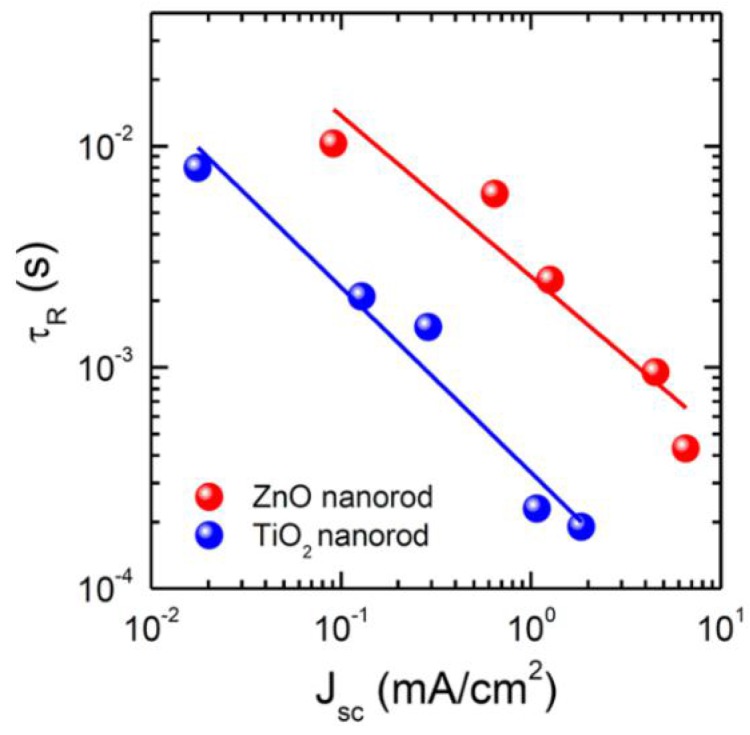
Comparison between time constant for charge recombination (τ_R_) as a function of light intensity, represented by photocurrent density, for ZnO and TiO_2_ NRs. Adapted with permission from Ref. [132]. Copyright 2014 American Chemical Society.

Hydrothermal growth of ZnO NRs is most commonly used compared to other methodologies like the galvanic cell-based methods [136], electrochemical methods [76,139,140], and magnetron sputtering [133] because of its flexibility in morphology tuning by adjusting conditions like growth time, growth temperature, precursor concentration, and addition of capping agents in the precursor [60,62,141]. In the hydrothermal growth of ZnO NRs, a thin seed layer of ZnO is deposited over the substrate by spin coating, sputtering, or chemical vapor deposition. Seeding of ZnO lowers the thermodynamic barrier by providing nucleation sites and also helps to improve the aspect ratio of the obtained rods [142]. The substrate with the seed layer is then kept in growth solution for the desired time. The growth solution is an aqueous solution of an alkaline reagent like sodium hydroxide or hexamethylenetetramine (HMTA) and a Zn^2+^ salt like Zn(NO)_3_ or ZnCl_2_. After synthesis, the ZnO NRs are washed and dried. The diameter of such NRs can be modified by changing the precursor solution while their length can be tuned by varying growth time [132]. Son et al. compared the performance of ZnO NRs and rutile TiO_2_ NRs as ETLs and found that ZnO NRs have a lower recombination rate compared to rutile TiO_2_ NRs, shown in Figure 11 [132].

**Figure 12:**
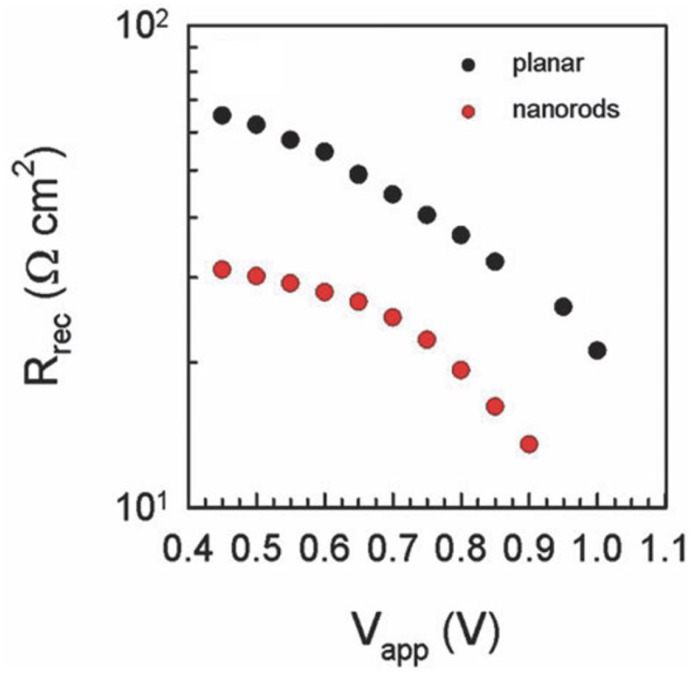
Comparison of recombination resistance of planar ZnO and ZnO nanorod-based perovskite solar cells. Adapted with permission from Ref. [130] with permission of The Royal Society of Chemistry.

In 2013, Bi et al. [55] compared the performance of ZnO NR ETLs with mesoporous TiO_2_ ETL in MAPbI_3_ solar cells with spiro-MeOTAD as the HTM. Cross-sectional FESEM images showed excellent infiltration of the ZnO NR ETL by the solution processed MAPbI_3_. The photocurrent density increased from 8.9 mA·cm^−2^ to 12.7 mA·cm^−2^ as the length of ZnO NRs was increased from 400 nm to 1000 nm due to increased harvesting of photons by a thicker perovskite coating of the NRs. Bi et al. [55] found the electron transport time of ZnO NRs to be faster than that in mesoporous TiO_2_ ETLs with the same thickness, but also observed that recombination in ZnO NRs due to back-electron transfer was higher than that for TiO_2_ ETLs. This caused a reduction in open circuit voltage, particularly with increasing ZnO NR length; hence ZnO-based solar cells exhibited a lower overall efficiency compared to mesoporous TiO_2_-based solar cells [55]. Further increase in the length of ZnO NRs caused a reduction in electron life time (τ_e_) and an increase in electron transport time (τ_tr_). Thus, like TiO_2_ NTs, and NRs, shorter ZnO NRs have a better electron transport property compared to longer NRs. Law et al. reported that the electron transport in ZnO NRs is faster than that in compact ZnO ETLs [143]. Kumar et al. compared the performance of planar ZnO and ZnO NRs for ETL in perovskite solar cells and found that the recombination resistance of ZnO NRs is lower compared to that of planar ZnO (as shown in Figure 12), which resulted in a decrease in open circuit voltage of nanorod based solar cells [130].

### 6.4. Non-Oxide 1D-Nanostructures

**Figure 13:**
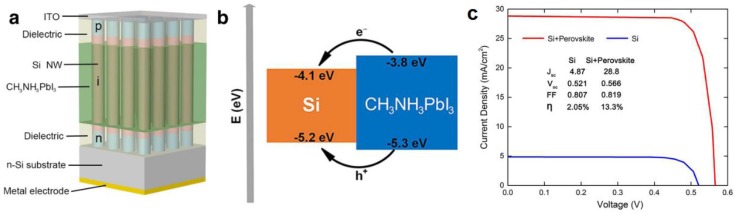
(**a**) Schematic diagram of the Si NW array/perovskite hybrid solar cell; (**b**) Band alignment scheme for the Si/MAPbI_3_ heterostructure and (**c**) *J–V* characteristics of a Si NW array solar cell and a Si NW array/perovskite hybrid solar cell. Adapted with permission from Ref. [145] under the terms of the Creative Commons Attribution 4.0 International License.

In 2015, Sargent and colleagues [144] reported the formation of a two dimensional superlattice consisting of PbSe nanowires in a halide perovskite matrix. PbSe NWs, instead of the more typical colloidal quantum dots, were obtained in a hot injection synthetic process due to directional fusion of the PbSe nanocrystals mediated by halide perovskite capping ligands. Furthermore, the separation of the nanowires was found to be equal to the lattice spacing of the corresponding perovskite (butylammonium lead iodide). While optical spectra clearly indicated harvesting of photons of wavelengths up to 1250 nm due to absorption from both the PbSe and perovskite phases, a solar cell was not demonstrated [144]. In a different report, Yan et al. [145] reported the infiltration of a Si nanowire array with MAPbI_3_ as shown in Figure 13. Here the *n*- and *p*-type sections of the Si NW functioned as the ETL and HTL respectively, while the halide perovskite was used purely as a light absorber that injected both the photogenerated electrons and holes into the Si NWs. 

## 7. Exploitation of Nanophotonic Effects in 1D-ETLs 

**Figure 14:**
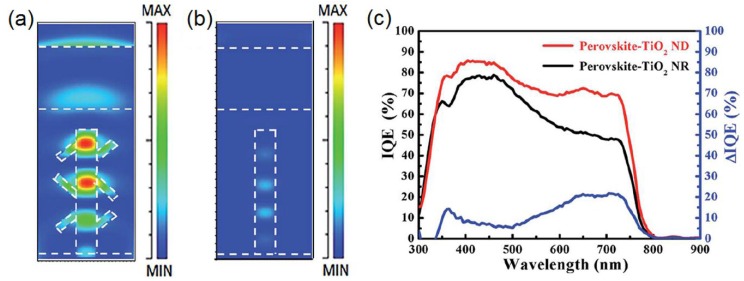
FDTD simulation results showing the simulated EM energy density distribution in MAPbI_3_-TiO_2_ NR/ND solar cells after pulse EM waves passing through: (**a**) perovskite–TiO_2_ nanodendrites (wavelength of 650 nm); (**b**) perovskite–TiO_2_ NRs (wavelength of 650 nm) and (**c**) IQE spectra of perovskite–TiO_2_ NR and perovskite–TiO_2_ ND solar cells. Adapted with permission from the Royal Society of Chemistry, original graphs appeared in Ref. [152].

The principal efficiency-limiting processes in state-of-the-art HPSCs (*η*~ 20%) are [146]: (i) Trap-assisted non-radiative recombination which increases the entropy of incident light and also necessitates a higher dark current density in order to meet the requirements of a black body in thermal equilibrium, which in turn decreases *V_oc_*; (ii) incomplete harvesting of near band-edge photons, resulting in suboptimal *J_sc_*; (iii) non-uniform splitting of the quasi-Fermi levels of electrons and holes through the cell and; (iv) reflection losses. It is noteworthy that 1D-ETLs are equipped to address each of the above limitations through exploitation of nanophotonic effects. Optimizing light-trapping alone could enable efficiencies of 24% to be obtained in a CH_3_(NH_2_)_2_PbI_3_ solar cell with a 180 nm effective thickness of the perovskite layer, while addressing all of the aforementioned limitations would enable efficiencies of up to 31% to be realized using a 200 nm active layer thickness [146,147]. Yet, research on nanophotonic engineering of halide perovskite solar cells significantly lags advances in materials processing and interface optimization. In 2011, Yeh et al. [148] demonstrated an increase in the efficiency of a single junction GaAs solar cell from 14.5% to 19.1% through the use of a graded antireflection coating external to the solar cell that included an array of vertically oriented, hexagonal ZnO NRs approximately 150 nm in width and 1.5 μm in height. The dramatic suppression of the reflectance by the nanostructured antireflection coating enabled a nearly 50% increase in the external quantum yield of photons in the 600–850 nm spectral range [148]. Similar strategies using ZnO NRs have been studied in amorphous silicon and crystalline silicon solar cells to decrease reflectance losses [149,150]. ZnO NRs with a high haze ratio due to Mie scattering effects have been used in *n*-ZnO/*p*-Cu_2_O heterojunction solar cells to maximize light trapping in the Cu_2_O absorber [151]; however an optimal ratio of forward scattering to backscattering is essential to achieve increases in the net photoconversion efficiency. A similar strategy involving branched TiO_2_ NRs (so-called nano-dendrites or ND) has been demonstrated to achieve modest improvements in the performance of HPSCs through enhancement of light trapping as shown in Figure 14 [152]. Cui et al. [153] demonstrated that a nanostructured active layer could achieve a fundamentally larger *V_oc_* than planar layers through both increased quasi-Fermi level splitting and enhanced radiative outcoupling efficiencies. 

## 8. Doping and Surface Modification

The electrical and optical characteristics of semiconductors may be modified through additives in the form of dopants. For example, TiO_2_ may be modified through the substitution of ions such as Al^3+^, Mg^2+^, Nb^5+^, Sn^4+^, or Y^3+^. Each of these dopants leads to different effects in the resulting photovoltaic device. Mg^2+^ has been demonstrated to increase the *V_oc_* of solar cells due to an elevated conduction band edge and suppressed recombination [154]. Nb^5+^ doping is able to fill trap states in TiO_2_ and improve electron transport properties [155,156,157], while Sn^4+^ doping has the potential to drastically increase the electron mobility of the ETL [158]. Yang et al. attempted to improve the charge transport efficiency of TiO_2_ NRs by doping it with Nb and was successfully able to improve the PCE of perovskite solar cell by 50%, as doping with Nb increased the recombination resistance at the perovskite/TiO_2_ NR interface and reduced the series resistance [113]. Similarly, Zhang et al. doped the NRs with Sn and observed an increase in bandgap from 3.0 eV to 3.04 eV. Doping with Sn also helped to reduce the series resistance and hence boost the PCE of the solar cell by 67% compared to a device using non-doped TiO_2_ NRs [140]. In another report, Li et al. deposited SnO_2_-Sb_2_O_3_ composite by spin coating and observed a reduction in charge transport resistance in the TiO_2_ NRs, and a 19% improvement in PCE compared to non-doped TiO_2_ NRs [159]. Another way to improve the charge transport properties of TiO_2_ NRs is to passivate them by the addition of another layer of TiO_2_ through a TiCl_4_ treatment. Such a TiCl_4_ treatment improves the roughness factor, charge transport efficiency, and crystallinity of the ETL [160,161,162]. Tao et al. studied the effect of TiCl_4_ treatments on TiO_2_ NRs, and found that they cause a reduction in the series resistance of resultant solar cells, improving the current density and PCE [163]. Using a similar approach, Mali et al. used atomic layer deposition to deposit 5 nm of TiO_2_ over hydrothermally grown TiO_2_ NRs to improve the charge transfer efficiency of the ETL [115]. Similar to TiO_2_, ZnO may be doped. In 2014, Dong et al. reported that by doping ZnO nanorods with a small concentration of Al^3+^, the recombination resistance increased significantly, resulting in improvement in the open circuit voltage of the solar cell and hence improving the PCE from 8.5% to 10.5% [131]. Mahmood et al. doped ZnO nanowire with nitrogen by adding ammonium acetate in growth solution. Doping with nitrogen enhanced charge-carrier concentration to improve the electron transport property of nanowire. They reported that the performance of solar cell depends upon the aspect ratio and length of nanowire. Chen et al. introduced nickel (II) acetate in growth solution of ZnO. Ni-doping of ZnO facilitates more align morphology, better conductivity and higher electron mobility. By doping ZnO nanowire with 2% of Ni device performance was boosted to 12.77% from 10.37% with MAPbI_3_ as the active layer [164].

Surface modification of one dimensional nanostructured ETLs can play an important role in improving HPSC performance. Such modification can lead to changes in perovskite deposition and energy band levels, as well as modify the processes of charge separation and charge collection. For example, Xu et al. introduced the fullerene derivative PCBM to ZnO NRs [138]. Owing to the rougher surface created by the PCBM on the ZnO NR array, they could achieve a greater perovskite loading. Furthermore, as PCBM has its energy levels between that of CH_3_NH_3_PbI_3_ and ZnO, electrons can cascade from the conduction band of one material to the next resulting in improved charge separation and faster injection of photogenerated electrons into the ZnO ETL, and overall recombination is reduced leading to improved device performance. For TiO_2_ ETLs, most surface modification work has been performed on mesoporous and planar TiO_2_ ETLs as most research efforts in general have been directed towards devices using those types of ETLs. However, in many cases similar surface modification treatments using one-dimensional nanostructured ETLs could also be expected to achieve superior results, and may highlight an important future research area. For example, mesoporous TiO_2_ has been functionalized with amino acids such as glycine to allow for better perovskite crystal growth, passivation of surface traps and concomitant higher photocurrent densities [165,166]. Self-assembled monolayers of 4-aminobenzoic acid (PABA) have also been prepared on top of mesoporous TiO_2_, which led to a lowered defect density and reduced surface traps [167]. Thiols have also been used as an interface modifier between mesoporous TiO_2_ and perovskite to facilitate the growth of larger perovskite grains and improved charge transfer between perovskite and the ETL [168]. High densities of surface states are known to limit carrier transport in TiO_2_ NR and NT arrays [73,169,170]. Recently, our group demonstrated that the surface passivation of hydrothermally grown TiO_2_ NR arrays by octadecylphosphonic acid (ODPA) resulted in a 2–3 order increase in the electron mobility of the NRs [171]. 

## 9. Conclusions

ETLs play an important role in the overall performance of perovskite solar cells, facilitating electron transport and limiting the geminate recombination of electrons and holes. Another role that ETLs could play but one that has not been explored sufficiently, is that of a photon management layer (PML), since at the high performance end it is losses related to the management of photons that limit the achievement of efficiencies close to the single junction theoretical limit. Electron transfer between perovskite/ETL and ETL/electrode is also critical when considering which ETL to use. It is important to choose an optimum thickness for the ETL to achieve superior photovoltaic performance of perovskite solar cells—too thin can result in insufficient light harvesting and recombination to occur, while too thick can hamper electron flow. One-dimensional electron transport layers have increasingly been the subject of study as perovskite solar cells themselves have risen to prominence. While one-dimensional nanostructures made of TiO_2_ and ZnO have been the primary subjects of study, other materials such as SnO_2_ and WO*_x_* have also been investigated as replacements. 1D nanostructures offer superior electron transport properties, with an electron diffusion lengths and charge collection efficiencies larger than the nanoparticle-based ETLs. Electron-hole recombination may be further reduced by doping and use of hybrid ETLs comprised of multiple materials. There are still many challenges that need to be addressed before one-dimensional nanostructure-based perovskite solar cells can achieve their full potential. Infiltration and surface passivation need to be improved, as both processes when suboptimal, result in lower solar cell efficiencies. The challenge of optimal infiltration can be at least partially solved through improved perovskite deposition processes. The dimensions of one-dimensional nanostructures still need to be optimized to balance competing processes. Finally, a deeper understanding and exploitation of nanophotonic phenomena to improve the management of photons are still needed to devise solar cell architectures with higher efficiencies.

## Figures and Tables

**Table 1:** Summary of solar cell performance reported to date for one-dimensional TiO_2_ ETLs.

Year	Device Structure	*V_OC_* (V)	*J_SC_* (mA·cm^−2^)	FF	PCE (%)	Ref.
2013	FTO|TiO_2_ BL|TiO_2_ NR|MAPbI_3_|Spiro|Au	0.82	10.1	0.59	4.87	[110]
2013	FTO|TiO_2_ BL|TiO_2_ NR|MAPbI_3_|Spiro|Au	0.96	15.6	0.63	9.40	[111]
2014	FTO|TiO_2_ BL|TiO_2_ NR|MAPbI_3_|Spiro|Au	0.77	22.3	0.68	11.7	[112]
2014	FTO|TIO_2_ BL|TIO_2_ NT| MAPbI_3_|Spiro	0.63	17.9	0.58	6.52	[57]
2014	FTO|TiO_2_ BL|Nb-TiO_2_ NR|*MAPbI_x_Br_3-x_*|Spiro|Au	0.87	16.5	0.52	7.50	[113]
2014	FTO|TiO_2_ BL|Sn-TiO_2_ NR|MAPbI_3_|Spiro|Ag	0.74	14.9	0.52	6.31	[114]
2015	FTO|TiO_2_ BL|TiO_2_ NR/Layer|MAPbI_3_|Spiro|Au	0.95	19.8	0.72	13.5	[115,116]
2015	FTO|TiO_2_ NT|MAPbI_3_|Spiro|Au	1.07	22.6	0.64	14.8	[105]
2015	FTO|TiO_2_ BL|TIO_2_ NT|MAPbI_3_|Au	0.67	19.6	0.37	5.00	[109]
2015	Ti|TiO_2_ NT|MAPbI_3_|CNT Film|Spiro	0.99	14.4	0.68	8.31	[108]
2016	FTO|TiO_2_ BL|TiO_2_ NR|MAPbI_3_|Spiro|Au	1.04	22.9	0.76	18.2	[116]
2016	FTO|TiO_2_ NT|MAPbI_3-*x*_Ac*_x_*|Ag	1.06	20.5	0.7	15.2	[107]
2017	FTO|TiO_2_ BL|TiO_2_ NT network|MAPbI_3_|Ag	0.88	24.8	0.63	13.8	[106]

**Table 2:** Summary of solar cell performance reported to date for one dimensional ZnO ETLs.

Year	Device Structure	*V_OC_* (V)	*J_SC_* (mA·cm^−2^)	FF	PCE (%)	Ref.
2013	FTO|ZnO NW|MAPbI_3_|Spiro|Ag	0.68	12.7	0.58	5.00	[55]
2013	FTO|ZnO SL|ZnO NW|MAPbI_3_|Spiro|Au	1.02	17.0	0.51	8.90	[130]
2014	FTO|ZnO SL|ZnO NW|AZO|MAPbI_3_|Spiro|Au	0.90	19.8	0.60	10.7	[131]
2014	FTO|ZnO SL|ZnO NW|MAPbI_3_|Spiro|Au	0.99	20.1	0.56	11.1	[132]
2014	FTO|ZnO NR|MAPbI_3_|Spiro|MoO_3_|Ag	1.04	22.4	0.57	13.4	[133]
2014	FTO|TiO_2_|ZnO SL|ZnO NW|MAPbI_3_|Spiro|Ag	0.93	18.0	0.62	10.4	[134]
2014	FTO|ZnO NR|PEI|MAPbI_3_|Spiro|Ag	0.97	21.7	0.70	16.2	[60]
2015	FTO|ZnO NW|TiO_2_ core shell|MAPbI_3_|Spiro|Au	1.00	22.0	0.70	15.4	[61]
2016	FTO|ZnO SL|ZnO NR|MAPbI_3_|Spiro|Au	0.68	21.6	0.62	9.06	[135]
2016	FTO|ZnO NR|SnO_2_|Spiro|Ag	0.90	23.3	0.57	12.2	[76]
2016	AZO|ZnO NR|MAPbI*_x_*Cl_3-*x*_|Carbon	0.86	14.9	0.28	3.62	[136]
2016	FTO|ZnO-TiO2 NW|ZrO_2_|MAPbI_3_|Carbon	0.96	14.8	0.58	8.24	[137]
2016	ITO|Ni-ZnO NW|PCBM|(MA)*_x_*(GA)_1-*x*_PbI_3_|P3HT|Au	0.83	23.7	0.70	13.8	[34]
2016	FTO|ZnO SL|ZnO NW|PCBM| MAPbI_3_|Spiro|Au	0.96	22.1	0.55	11.7	[138]

**Table 3:** Summary of reported device performances from solar cells incorporating ETLs containing composites and unusual morphologies.

Year	Device Structure	*V_OC_* (V)	*J_SC_* (mA·cm^−2^)	FF	PCE (%)	Ref.
2016	FTO|ZnO-TiO2 NW|ZrO_2_|MAPbI_3_|Carbon	0.96	14.8	0.58	8.24	[137]
2016	ITO|Ni-ZnO NW|PCBM|(MA)*_x_*(GA)_1-*x*_PbI_3_|P3HT|Au	0.83	23.7	0.70	13.8	[34]
2015	FTO|ZnO NW|TiO_2_ core shell|MAPbI_3_|Spiro|Au	1.00	22.0	0.70	15.4	[61]
2014	FTO|TiO_2_|ZnO SL|ZnO NW|MAPbI_3_|Spiro|Ag	0.93	18.0	0.62	10.4	[134]
2014	FTO|TiO_2_ BL/SL|Nb-TiO_2_ NR|MAPbI*_x_*Br_1-*x*_|Spiro|Au	0.87	16.5	0.52	7.50	[113]
2014	FTO|TiO_2_ SL|Sn-TiO_2_ NR|MAPbI_3_|Spiro|Ag	0.74	14.9	0.52	6.31	[114]
2015	FTO|TiO_2_ BL|TiO_2_ NR/Layer|MAPbI_3_|Spiro|Au	0.95	19.8	0.72	13.5	[115]
2015	FTO|WO_3_ BL|WO_3_ NR|TiO_2_|MAPbI_3_|Spiro|Ag	0.86	15.00	0.70	9.10	[38]
